# Comparative analysis of the bronchoalveolar microbiome in Portuguese patients with different chronic lung disorders

**DOI:** 10.1038/s41598-021-94468-y

**Published:** 2021-07-22

**Authors:** Susana Seixas, Allison R. Kolbe, Sílvia Gomes, Maria Sucena, Catarina Sousa, Luís Vaz Rodrigues, Gilberto Teixeira, Paula Pinto, Tiago Tavares de Abreu, Cristina Bárbara, Júlio Semedo, Leonor Mota, Ana Sofia Carvalho, Rune Matthiesen, Patrícia Isabel Marques, Marcos Pérez-Losada

**Affiliations:** 1 i3S - Instituto de Investigação e Inovação em Saúde, Universidade do Porto, Rua Alfredo Allen, 208, 4200-135 Porto, Portugal; 2 Institute of Molecular Pathology and Immunology, University of Porto (IPATIMUP), Porto, Portugal; 3 Computational Biology Institute, Department of Biostatistics and Bioinformatics, Milken Institute School of Public Health, The George Washington University, Washington, DC USA; 4 Pneumology Department, Centro Hospitalar de São João (CHSJ), Porto, Portugal; 5 Department of Pneumology, Unidade Local de Saúde da Guarda (USLGuarda), Guarda, Portugal; 6 Department of Pneumology, Centro Hospitalar Do Baixo Vouga (CHBV), Aveiro, Portugal; 7 Unidade de Pneumologia de Intervenção, Hospital Pulido Valente, Centro Hospitalar Universitário Lisboa Norte (CHULN), Lisbon, Portugal; 8 Instituto de Saúde Ambiental, Faculdade de Medicina da Universidade de Lisboa, Lisbon, Portugal; 9 Computational and Experimental Biology Group, CEDOC, Faculdade de Ciências Médicas, Universidade Nova de Lisboa, Lisbon, Portugal; 10 CIBIO-InBIO, Centro de Investigação em Biodiversidade e Recursos Genéticos, Universidade do Porto, Campus Agrário de Vairão, Vairão, Portugal

**Keywords:** Respiratory tract diseases, Microbiome

## Abstract

The lung is inhabited by a diverse microbiome that originates from the oropharynx by a mechanism of micro-aspiration. Its bacterial biomass is usually low; however, this condition shifts in lung cancer (LC), chronic obstructive pulmonary disease (COPD) and interstitial lung disease (ILD). These chronic lung disorders (CLD) may coexist in the same patient as comorbidities and share common risk factors, among which the microbiome is included. We characterized the microbiome of 106 bronchoalveolar lavages. Samples were initially subdivided into cancer and non-cancer and high-throughput sequenced for the 16S rRNA gene. Additionally, we used a cohort of 25 CLD patients where crossed comorbidities were excluded. Firmicutes, Proteobacteria and Bacteroidetes were the most prevalent phyla independently of the analyzed group. *Streptococcus* and *Prevotella* were associated with LC and *Haemophilus* was enhanced in COPD versus ILD. Although no significant discrepancies in microbial diversity were observed between cancer and non-cancer samples, statistical tests suggested a gradient across CLD where COPD and ILD displayed the highest and lowest alpha diversities, respectively. Moreover, COPD and ILD were separated in two clusters by the unweighted UniFrac distance (*P* value = 0.0068). Our results support the association of *Streptoccocus* and *Prevotella* with LC and of *Haemophilus* with COPD, and advocate for specific CLD signatures.

## Introduction

The human lung was originally thought to be a sterile organ; however, it is now accepted to harbor a complex community of microorganisms referred to as the lung microbiome^1,2^. Notably, the lung microbiome has been shown to be remarkably similar to the oropharyngeal microbiota due to the physiological mechanism of micro-aspiration, which facilitates bacterial dissemination from the upper airways into the lower respiratory tract^2,3^. In healthy lungs there is indeed a bidirectional flow of microbes with a steady equilibrium between immigration and elimination through mucociliary clearance^2–5^. Although bacterial biomass in the lung is maintained at a low concentration, it displays a remarkable microbiological diversity^2,4^. According to different studies, the lung microbiome is dominated by the phyla Firmicutes, Proteobacteria and Bacteroidetes, and the genera *Prevotella*, *Veillonella* and *Streptococcus*^2,4^. Bacterial burden, however, frequently fluctuates in chronic lung disorders (CLD), particularly during acute disease stages (exacerbations) and life-threatening complications (e.g., septicemia)^4–6^.

CLD encompass several airway pathologies, such as chronic obstructive pulmonary disease (COPD), interstitial lung disease (ILD) and lung cancer (LC). COPD is a common CLD and a leading cause of morbidity and mortality worldwide, and is associated mainly with cigarette smoking as well as several indoor and outdoor hazards^7,8^. Nowadays, COPD is characterized by persistent respiratory symptoms and airflow limitation that is not fully reversible as assessed by lung function tests (spirometry). Small airways obstruction (e.g., bronchitis and bronchiolitis) and loss of lung parenchyma (emphysema) are major underlying causes of COPD and usually coexisting at different scales^7,8^. The COPD microbiome shows high heterogeneity during stable phases and undergoes notable shifts toward Proteobacteria (mostly *Moraxella* and *Haemophilus*) during exacerbations and advanced disease stages^9,10^.

ILD comprises a wide group of disorders sharing common features of enhanced fibrosis. ILD affects primarily the lung interstitium and can be triggered by a plethora of environmental and/or immunological exposures^2,11^. Idiopathic pulmonary fibrosis (IPF) represents a paradigmatic example of ILD in which lung architecture is seriously compromised by the accumulation of extensive scar tissue of unknown etiology. Other less prevalent and scrutinized ILD include sarcoidosis, a systemic disorder characterized by idiopathic appearance of granuloma that affects predominantly the lung, and hypersensitivity pneumonitis (HP) a complex syndrome resulting from a negative reaction to antigen inhalation (e.g., non-tuberculosis mycobacteria).

Concerning the ILD microbiome, most of the available data is related to IPF. Those studies showed that in stable patients there is a two-fold increase in bacterial load coupled with diversity loss mostly due to an overgrowth of potentially pathogenic genera (*Streptococcus, Neisseria* and *Haemophilus*)^2,12^. In addition, during acute IPF exacerbations, microbial abundance was found to increase and, as in COPD, a boost in Proteobacteria prevalence was also observed^13^.

Finally, LC is the most commonly diagnosed and lethal of all cancers. Like COPD, LC is also directly correlated with the tobacco epidemic and several air pollutants (e.g., asbestos and biomass burning)^14,15^. LC is classified into different histological types being the most prevalent the non-small cell lung cancer (NSCLC), which can be further subdivided into distinct carcinomas where the most common are the adenocarcinoma (ADC) and the squamous cell carcinoma (SCC). Few microbiome studies have discriminated between LC subtypes, but overall, they seem to reveal a reduction in microbial diversity coupled with significant changes in some bacterial genera (e.g., S*treptococcus and Veillonella* enrichment) during LC. Remarkably, those alterations seem to be perceptible not only in tumor sites, but also in distant non-cancerous regions of the lung^16–18^. Moreover, according to a recent study, lung microbiota seems to differentially impact SCC patient survival, either because bacteria (Enterobacteriaceae) cause non-cancer complications of infectious nature, or because they enhance inflammatory pathways and carcinogenic events^19^.

In LC as well as in COPD and ILD, inflammatory processes are often upregulated^20,21^. It is thought that microbiome dysbiosis may play a role in the activation and perpetuation of inflammatory processes, which ultimately may impact biological networks and disease progression^4,5,22^. Furthermore, these CLD are proposed to be linked by common mechanisms of pathogenesis, where pulmonary emphysema and fibrosis have been recognized as critical lung injuries often preceding malignant transformation. Moreover, COPD and ILD may coexist with LC in the same individual as comorbidities, which leads to worse outcomes of the disease^20,23,24^.

Therefore, to understand the etiology of CLD, it is crucial to disentangle the contribution of the lung microbiome to each disorder, in particularly to LC, which is far less studied when compared to COPD and ILD. Furthermore, it is also fundamental to assess whether lung microbiotas are influenced by different risk factors, such as smoking history, patient age, gender or even disease type and their overlap. To this end, we have combined 16S rRNA amplicon next-generation sequencing with bioinformatics to first characterize the lung microbiome of 89 Portuguese individuals with and without LC, regardless of their histological type and COPD or ILD co-occurrence. Additionally, we have compared the lung microbiomes of 25 CLD patients diagnosed with LC, COPD or ILD and controlled for the absence of crossed comorbidities.

## Material and methods

### Ethics approval and consent to participate

Sample collection for research purposes was authorized by the ethical committees of participating institutions: *Comissão de Ética para a Saúde* (CES) *Centro Hospitalar São João* (#95_14)*;* CES *Centro Hospitalar Baixo Vouga (*#054031*),* and the Ethics Committees from the *Centro Hospitalar Lisboa Norte* and the National Health Institute Dr. Ricardo Jorge (DIRCLN-8ABR2014-130)*.* Informed consent was obtained from all participants and patient samples and data were treated anonymously. The study was conducted in accordance with ethical guidelines and regulations for Human research and the Helsinki Declaration.

### Samples

Bronchoalveolar lavage fluid (BALF) samples were collected by pulmonologists from individuals subjected to a bronchoscopy for evaluation of lung disease at three hospitals in Portugal: *Centro Hospitalar São João* (CHSJ), Porto; *Centro Hospitalar Baixo Vouga* (CHBV), Aveiro; and *Hospital Pulido Valente—Centro Hospitalar Universitário Lisboa Norte* (CHULN), Lisbon. Sample collection targeted affected lung segments and was carried out as previously described^19,25^. Briefly, BALF samples had a minimum volume of 15 mL (0.9% saline solution) and were initially stored by pulmonologists at − 20 to 4 °C according to the facilities available at the participating hospitals. Samples were then transported on ice to research centers where they were stored at − 80 °C until needed. Overall, we collected 106 samples to address two main goals: (1) compare the lung microbiome of LC cases with other non-cancerous patients and (2) contrast the lung microbiome of LC patients with those of COPD and ILD patients. Towards the first goal, we sampled 49 patients with a positive cancer diagnosis (LC) (regardless of histological type and the presence of other known comorbidities such as COPD or ILD), and 40 patients with a negative cancer diagnosis (non-LC; Supplementary Table 1). Moreover, we did not include in the non-LC group any subject with a primary diagnosis of COPD or ILD. No healthy controls were collected due to bronchoscopy invasiveness and risk of complications.

**Figure 1:**
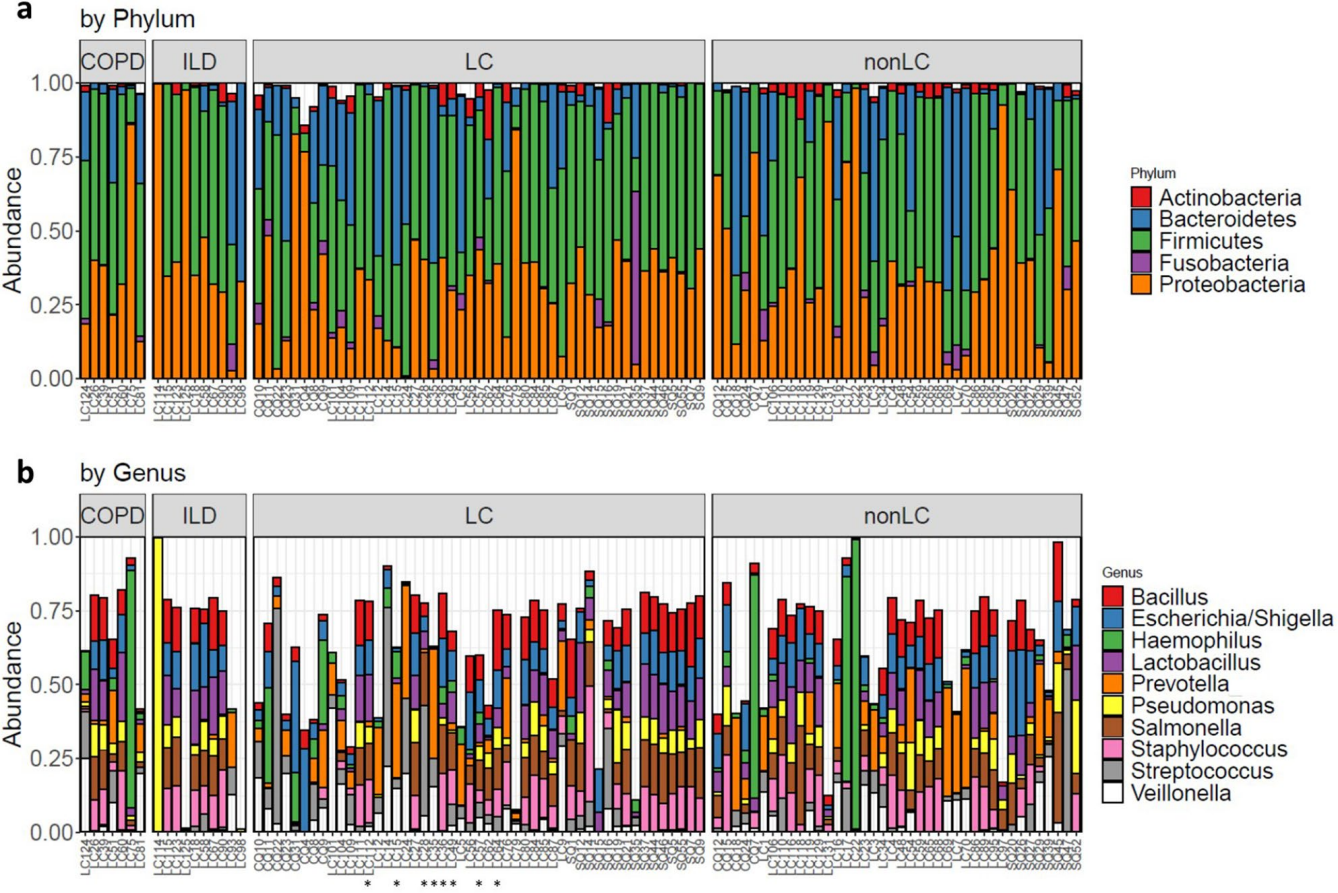
Microbial profiles of most abundant (> 1%) phyla (**a**) and genera (**b**) per individual BALF sample. Comorbidity controlled groups: COPD (N = 7) and ILD (N = 10), and LC (N = 49) and non-LC (N = 40) samples are indicated. Samples included in the LC* (N = 8) controlled group are marked with asterisks (*).

To address our second goal, we selected three homogenous patient groups with a single CLD diagnosis (controlled for other comorbidities): LC (N = 8), COPD (N = 7) and ILD (N = 10). LC patients were included in the comparison above (Supplementary Table 1; Table 2). For simplicity, this subset of LC samples will be designated from this point forward as LC*. This is also indicated in Fig. 1. To our knowledge, none of the patients included in this study had a record of acute exacerbations at the time of sampling.

### Lung microbiota 16S rRNA screening and analysis

DNA extraction from BALF (200–250 µL) was performed using DNA Mini kit (Qiagen) according to manufacturer’s instructions for capturing bacterial DNA in body fluids. We amplified and sequenced a fragment of ~ 250 bp of the 16S rRNA gene covering the V4 region using the dual-index sequencing strategy described in Kozich et al.^26^. Sequencing was performed using the next-generation sequencing Illumina MiSeq platform at the GWSPH Genomics Core Facility. We sequenced both negative controls and mock communities (reference samples with a known composition) to detect potential contaminating microbial DNA in reagents and measure sequencing error rate. No evidence of contamination was found and our sequencing error rate was low. Sequence data have been deposited in GenBank under BioProject PRJNA742244.

16S rRNA–V4 amplicon sequence variants (ASV) in each sample were inferred using dada2 version 1.16^27^. Exact sequence variants provide a more accurate and reproducible description of amplicon-sequenced communities than is possible with operational taxonomic units (OTUs) defined at a constant level (97% or other) of sequence similarity^27^. Reads were filtered using standard parameters, with no uncalled bases, maximum of 2 expected errors, and truncating reads at a quality score of 2 or less. Forward and reverse reads were truncated after 225 and 100 bases, respectively. The standard dada2 pipeline was then applied to perform ASV inference, merge paired reads and identify chimeras. Taxonomic assignment was performed against the Silva v132 database using the implementation of the RDP naive Bayesian classifier available in the dada2 R package^28,29^. ASV sequences were aligned using MAFFT^30^ and used to build a tree with FastTree^31^. The resulting ASV tables and phylogenetic tree were imported into phyloseq^32^ for further analysis.

We normalized our samples using the negative binomial distribution as recommended by McMurdie and Holmes^33^ and implemented in the Bioconductor package DESeq2^34^. This approach simultaneously accounts for library size differences and biological variability and it has increased sensitivity in small and homogeneous datasets with less than 20 samples per group^35^. Microbial normalized counts generated this way are referred to as taxon abundances throughout the text. Taxonomic and phylogenetic alpha-diversity were estimated using Chao richness and Shannon, ACE, Simpson, Fisher and Phylogenetic (Faith’s) diversity indices. Beta-diversity was estimated using phylogenetic Unifrac (unweighted and weighted), Bray–Curtis and Jaccard distances. Dissimilarity between samples was explored using principal coordinates analysis (PCoA).

Significant associations between alpha-diversity indices and taxon abundances and lung disorders and covariables (clinical history, age and sex) were assessed using the Mann–Whitney-Wilcoxon Test. Beta-diversity indices were compared using permutational multivariate analysis of variance (adonis) as implemented in the vegan R package^36^. We applied the Benjamini–Hochberg method at alpha = 0.05 to correct for multiple hypotheses testing^37,38^. Effect sizes were calculated using Cohen’s d_s_ estimator for unequal group sizes^39^. All the analyses above were performed in R^40^ and RStudio^41^.

## Results

### Subjects biodemographic and clinical characteristics

In our study LC patients averaged 65.6 years of age, 41 (83.7%) were men and 67.3% were reported as former or current smokers. NSCLC was the most prevalent cancer (51%) among these patients, with ADC and SCC subtypes representing 34.7% and 10.2% of cases, respectively. A small fraction of LC subjects was diagnosed with SCLC or with other rarer cancers types (14.3%) and for the remaining samples no cancer type classification was available (Table 1; Supplementary Table 1). Non-LC individuals were younger and averaged 59.5 years of age, 27 (54%) were men and 50% described as former or current smokers (Table 1; Supplementary Table 1). A heterogeneous array of respiratory conditions was reported for non-LC subjects, including many benign findings (22.5%) and several lung abnormalities such as hemoptysis and atelectasis (Table 1; Supplementary Table 1).

**Table 1:** Demographic and clinical data of the extended BALF dataset.

Variables	LC (N = 49)	Non-LC (N = 40)	*P* value*
**Age (yrs.** **±** **SD)**	65.6 **±** 11.4	59.5 **±** 12.7	0.01805
**Sex, men N (%)**	41 (83.7)	27 (54.0)	NS
**Smoking status N (%)**			
Smoker	16 (32.7)	10 (25.0)	NS
Former smoker	17 (34.7)	10 (25.0)	
Non-smoker	9 (18.4)	15 (37.5)	
Unknown	7 (14.3)	5 (12.5)	
**LC Diagnosis N (%)**			
NSCLC	25 (51.0)		NA
ADC	17 (34.7)		
SCC	5 (10.2)		
LCC	1 (2.0)		
Unknown	2 (4.1)		
SCLC	5 (10.2)		
Others	2 (4.1)		
Unknown	17 (34.7)		
**Non-LC Diagnosis N (%)**			
Hemoptysis		5 (12.5)	NA
Atelectasis		3 (7.5)	
Unaffected		2 (5.0)	
Benign findings		9 (22.5)	
Asthma		1 (2.5)	
Hamartoma		1 (2.5)	
Other		7 (17.5)	
Unknown		14 (35.0)	

*P values based on Welch's t-test or chi-square (normal distributed or categorical variables, respectively) for the comparison lung cancer (LC) and non-LC groups. NS—non-significant (*p* value > 0.05). NA—not applicable.

In the CLD comorbidity-controlled groups, the LC subset (LC*) averaged 58.5 years of age and comprised 7 NSCLC (5 ADC) and 1 SCLC types; five were men, and five had a history of heavy smoking (20–63 packs per year; PPY). The COPD group (mean age 56.7 years) included only moderate disease cases (GOLD 2), a single woman and four heavy smokers (38–120 PPY). Finally, the ILD group (mean age 62.9 years) included 3 HP, 2 sarcoidosis and a single IPF case, 7 patients were men and 6 were former smokers (Supplementary Table 1; Table 2).

**Table 2:** Demographic and clinical data of the comorbidity-controlled dataset.

Variables	LC* (N = 8)	COPD (N = 7)	ILD (N = 10)	*P *value*
**Age (yrs.** **±** **SD)**	58.5 **±** 14.1	56.7 **±** 13.9	62.9 **±** 12.3	NS
**Sex, men N (%)**	5 (62.5)	6 (85.7)	8 (80.0)	NS
**Smoking status N (%)**				
Smoker	3 (37.5)	2 (28.6)	2 (20.0)	NS
Former smoker	2 (25.0)	3 (42.6)	6 (60.0)	
Non-smoker	3 (37.5)	2 (28.6)	2 (20.0)	
**Pack Per Year**	37.6 **±** 18.2	64.5 **±** 37.4	35.7 **±** 17.7	NS^a^
**LC Diagnosis N (%)**				
NSCLC	7 (87.5)			NA
ADC	5 (62.5)			
Unknown	2 (25.0)			
SCLC	1 (12.5)			
**COPD Diagnosis N (%)**				
GOLD 2		5 (71.4)		NA
Unknown		2 (28.6)		
**ILD Diagnosis N (%)**				
HP			3 (30.0)	NA
Sarcoidosis			2 (20.0)	
IPF			1 (10.0)	
Other			4 (40.0)	

*P values based on Kruskal–Wallis test or chi-square (normal distributed or categorical variables, respectively) for the comparison of the three groups, lung cancer (LC), chronic obstructive pulmonary disease (COPD) and interstitial lung disease (ILD). HP- Hypersensitivity pneumonitis; NS—Non-significant (*p* value > 0.05); NA—Not applicable. ^a^—results based in pairwise Welch's t-tests.

#### Taxonomic characterization

In general, the microbiome analysis of BALF samples consistently showed Firmicutes, Proteobacteria, Bacteroidetes and Actinobacteria as the prevalent phyla across the five groups (Table 3; Fig. 1A). Similarly, the results obtained at the genus level indicated that abundant bacteria such as *Prevotella*, *Staphylococcus*, *Veillonella*, *Pseudomonas* and *Streptococcus* were also shared by the different groups (Table 3; Fig. 1B). Some inter-individual variability in microbial composition could be detected as suggested by a few outlier samples dominated by single genera (Fig. 1B). Interestingly, those samples were all subjected to microbiological culture testing, one being classified as negative (LC114), two positive and concordant with 16S rRNA results (LC75 with *Haemophilus* and LC125 with *Serratia*) and another positive but discordant (LC98; Supp. Table 1).

**Table 3:** Mean relative proportions of dominant phyla and genera (> 1%) identified in the different groups.

Taxon	Extended dataset		Comorbidity controlled dataset		
	LC	Non-LC	LC*	COPD	ILD
***Phyla***					
Firmicutes	47.11	40.30	48.75	49.69	39.30
Proteobacteria	31.35	37.94	30.17	34.71	45.06
Bacteroidetes	15.52	17.61	17.34	13.42	12.82
Actinobacteria	2.80	1.95	2.40	< 1.00	1.50
***Genera***					
*Prevotella*	6.09	8.32	**9.47**	4.63	**2.14**
*Escherichia/Shigella*	8.80	8.96	8.58	6.05	7.91
*Staphylococcus*	7.27	7.02	7.33	6.95	8.40
*Lactobacillus*	6.41	6.75	6.37	8.86	9.03
*Bacillus*	7.66	6.79	8.51	7.72	7.53
*Salmonella*	7.40	7.60	10.43	6.66	7.83
*Veillonella*	6.00	4.63	5.69	8.29	1.48
*Haemophilus*	3.21	7.06	2.90	**14.28**	** < 1.00**
*Pseudomonas*	3.56	5.09	2.63	5.16	14.18
*Streptococcus*	**7.45**	**3.94**	8.74	4.29	1.85

Taxa proportions with significant differences are highlighted in bold (*P* < 0.05).

*Escherichia/Shigella*, *Bacillus*, *Streptococcus* and *Salmonella* displayed the largest mean abundances in LC cases (Table 3). However, only *Streptococcus* diverged between LC and non-LC groups (Wilcoxon rank sum test; *p* value = 0.03852; Cohen’s d_S_ = 0.30). *Streptococcus, Prevotella, Salmonella* and *Escherichia/Shigella* were found as the most prevalent taxa in the comorbidity-controlled LC* group (Table 3), whereas *Prevotella* proportions separated LC* from ILD cases (Wilcoxon rank sum test;*p* value = 0.04405; Cohen’s d_S_ = 0.65).

Conversely, in the ILD group the most common taxa according to their mean abundances were *Pseudomonas*, *Lactobacillus, Staphylococcus,* and *Escherichia/Shigella*, (Table 3). Besides *Prevotella* (ILD vs. LC*), *Haemophilus* also varied significantly in the ILD versus COPD comparison (Wilcoxon rank sum test; *p* value = 0.005107; Cohen’s d_S_ = 0.74).

Finally, in the COPD controlled group *Haemophilus, Lactobacillus*, *Veillonella* and *Bacillus* comprised the most prevalent taxa (Table 3). No statistically significant differences were observed between COPD and LC* groups at the genus level.

Given that a strict definition of a common shared microbiome could not be applied to CLD comorbidity-controlled groups, we used instead a less constrained threshold, in which taxa were considered as common if present in at least 80% of the samples. With this approach Enterobacteriaceae (*Escherichia/Shigella* and *Salmonella*), *Staphylococcus*, *Streptococcus*, *Lactobacillus*, *Listeria* and *Bacillus* were recognized as members of a stable bacterial community shared across LC*, COPD and ILD.

#### Microbiota diversity

**Figure 2:**
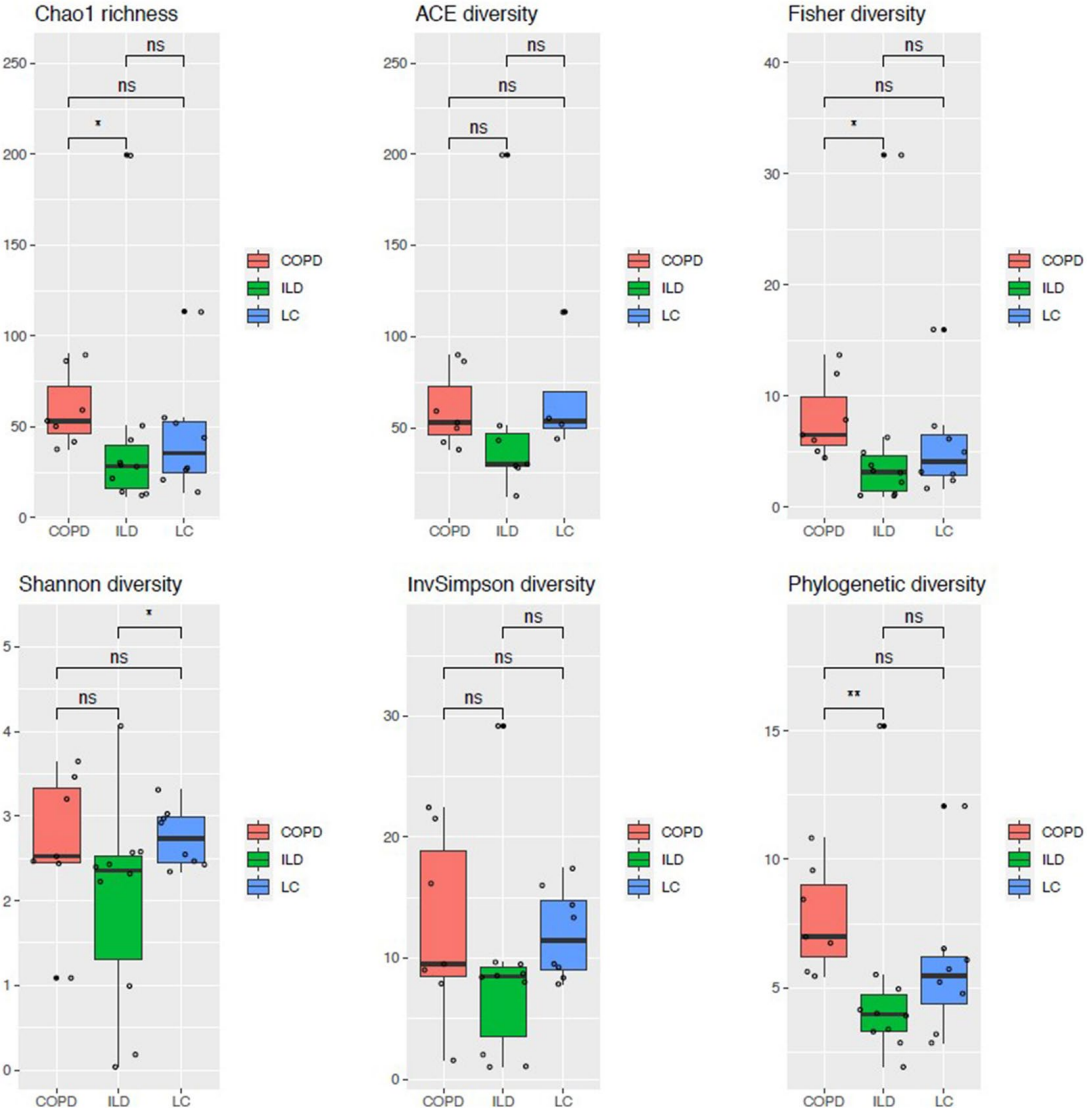
Alpha diversity of the CLD comorbidity-controlled dataset COPD (N = 7), ILD (N = 10) and LC* (N = 8) groups. Displayed estimates: Chao richness and Shannon, ACE, Inversed Simpson, Fisher and Phylogenetic (Faith’s) diversity indices. Significant *p-*values for pairwise group comparisons are indicated as (*) for* p* < 0.05 and (**) for *p* < 0.01. ns: non-significant.

**Figure 3:**
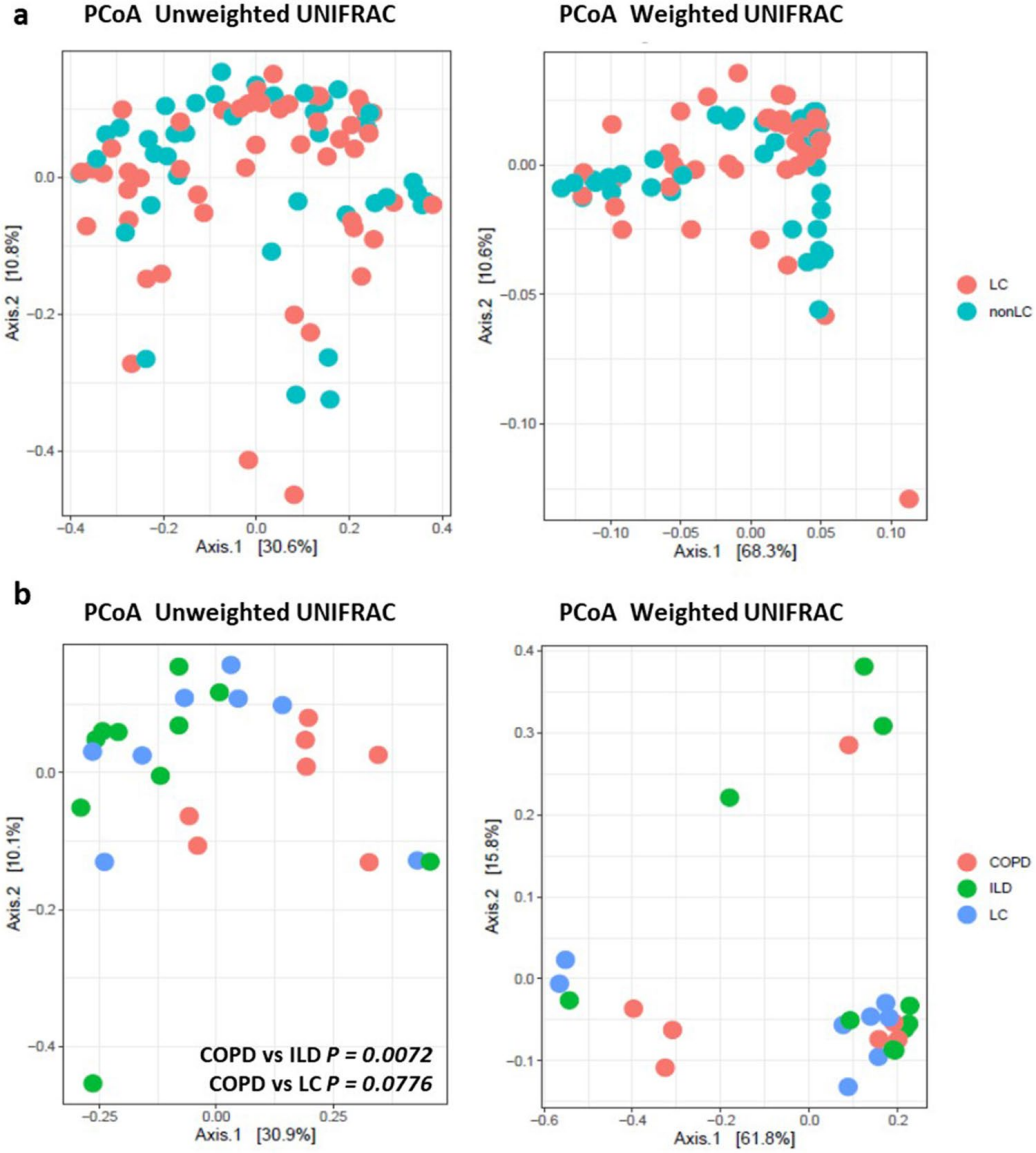
Beta diversity as shown by principle coordinate analysis of unweighted UniFrac distances and weighted UniFrac distances. (**a**) Extended dataset comprising LC (N = 49) and non-LC groups (N = 40); (**b**) Comorbidity controlled dataset including COPD (N = 7), ILD (N = 10) and LC* (N = 8) groups.

Alpha-diversity indices did not vary significantly between LC and non-LC groups (Supplementary Fig. 1; Supplementary Table 2). In contrast, CLD groups were found to differ, with COPD showing higher diversity than LC* and ILD. Statistically significant results were observed in Chao richness, Fisher and Phylogenetic diversity indices for COPD versus ILD (P_Chao_ = 0.0250; P_Fisher_ = 0.0185; and P_PD_ = 0.0068) and in Shannon diversity index for LC* versus ILD (*P* value = 0.0476; Fig. 2; Supplementary Table 2). PCoA plots did not reveal microbial structure (beta-diversity) for LC and non-LC groups, as suggested by the overlap of samples and non-significance of the adonis tests (Fig. 3A; Supplementary Fig. 2). On the other hand, among CLD types, PCoA plots showed some dissimilarities (Fig. 3B, Supplementary Fig. 2), with COPD versus ILD yielding significant differences in the unweighted Unifrac distance (adonis test* P* = 0.0072) and with COPD versus LC* showing a borderline, yet non-significant *p* value for the same statistic (P = 0.0776; Fig. 3B). In general, alpha- and beta-diversity were not affected by analyzed co-variables (data not shown), except for the LC* versus ILD comparison, in which smoking history could be associated with statistically significant differences in Bray–Curtis and Jaccard indices (*P* = 0.027 and *P* = 0.025, respectively; Supplementary Fig. 3).

## Discussion

Currently, few studies have attempted to simultaneously analyze the microbiome of distinct CLD considering their frequent co-occurrence in a single individual. In this study, we perform a characterization of BALF samples first stratified into LC and non-LC cases and then into three CLD groups (LC*, COPD and ILD) controlled for the absence of crossed comorbidities. Although, we found lung microbiome to be relatively stable among the studied groups, significant differences in the proportions of certain taxa were detected, suggesting a possible role for bacteria in the onset, progression and eventual outcome of distinct CLD. A recent comparison of bacterial communities from LC cases with an assorted group of lung disorders and with healthy controls (BALF samples also), presented no distinct profiles in alpha-diversity^18^. After stratifying samples into cancer and non-cancer types, the same study detected significant differences in beta-diversity tests^18^. We, however, found no significant differences in alpha- or beta-diversities between LC and non-LC groups, which could be related to the heterogeneous nature of the 49 cancerous samples compared, which comprise only 34.7% of ADC and 10.5% of SCC subtypes. Contrarily, Tsay et al. (2018) studied mostly ADC and SCC, representing 56.4% and 25.6% of the 39 cases analyzed, respectively^18^. Nonetheless, we did observe significant differences between other CLD groups. When controlling for comorbidities and comparing strict CLD phenotypes, COPD versus ILD displayed a remarkable divergence across both alpha- and beta- diversity indices (but not against LC*), indicating some community structuring by disease. According to our results, COPD communities are generally the most diverse and composed by a larger number of low-abundance taxa as suggested by the Chao index results. On the other hand, ILD cases show the lowest bacterial richness and diminished phylogenetic diversity. Interestingly, LC* samples, which overlapped with both COPD and ILD groups in most alpha indices, exhibited a stronger phylogenetic relatedness with ILD cases, as uncovered by the unweighted Unifrac distances.

Concerning bacteria differential abundances, *Streptococcus* was identified as significantly increased in the LC group compared to non-LC, whereas in the comorbidity-controlled dataset *Prevotella* was identified to be augmented in LC* when contrasted with ILD. In addition, in the COPD group, *Haemophilus* proportions were found to be higher than in ILD. All these genera, typically associated with the oral microbiome, have already been reported as prevalent taxa in affected lungs of CLD patients^12,16,18,42^.

Notably, *Streptococcus* and *Prevotella* proportions, which discriminate our LC cases from other assorted pathologies (LC vs. non-LC and LC* vs ILD groups, respectively), replicated to some extent Tsay et al. (2018) findings, where the same taxa were identified as good predictors of LC^18^. Therefore, our results may also support the association between the high prevalence of these microbes and lung carcinogenesis. To be more accurate, Tsay et al. (2018) demonstrated by means of in vitro studies that *Streptococcus* and *Prevotella* are able to induce the up-regulation of PI3K (phosphoinositide 3-kinase) and ERK (extracellular signal–regulated kinase) signaling pathways, which are associated with cancer transformation^18^. Importantly, *Streptococcus* is also a well-known pneumonia agent (*Streptococcus pneumoniae*), particularly among LC subjects. Moreover, *Streptococcus* has been shown to raise cytokine levels and promote diverse inflammatory responses through the activation of Toll-like receptors and by the degradation of extracellular matrix elements^16,43^.

Conversely, a high content of *Prevotella* in the airways has been correlated with enhanced concentrations of interleukin 17 (IL17), among other cytokines, and T helper 17 cells (Th17), underlying a status of subclinical lung inflammation seen also among healthy individuals^5,44,45^. Furthermore, in a recent study using bleomycin-induced mouse models of lung fibrosis, it was shown that a dysbiotic microbiome enriched *in Prevotella* could activate multiple pro-inflammatory and pro-fibrotic genes. These, in turn, were found to promote both lung immune cell infiltration and massive extracellular matrix deposition, ultimately leading to animal death in an IPF-like phenotype^46^. Once again, IL17 and Th17 cells were pinpointed as key drivers of inflammatory networks induced by *Prevotella* in mice^46^. In our study, we observed a decreased prevalence of *Prevotella* in the ILD cohort, but a higher prevalence in LC*. This may indicate a potential interaction between *Prevotella* and Th17 cells, which were hitherto shown to promote lung tumorigenesis^47^.

The detection of a higher proportion of *Haemophilus* in our COPD cases, a taxon frequently associated with acute exacerbations (*Haemophilus influenzae*), supports previous evidence for an early dysbiosis caused by this genus that can be observed even in stable phases of the disease^9,10^. Interestingly, *Haemophilus* has been described to provoke a more aggressive inflammatory response than *Prevotella*, as depicted by a fold increase in IL10, IL12 and IL23 cytokines^48,49^. In addition, it was correlated with the activation in the airways of the nuclear factor kappa beta (NF-KB) pathway and other inflammatory markers, such as IL1B and IL6, myeloperoxidase, and CXC-chemokine ligand 8. Moreover, *Haemophilus* is also capable of triggering other host responses that might be correlated with COPD pathogenesis, including the production of reactive oxygen species (ROS) and the formation of extracellular protease networks traps by both neutrophil and macrophage cells^50,51^.

The hypothesis of the microbiome fulfilling a pivotal role in CLD seems quite plausible if considering the negative effects of the aforementioned bacteria in lung biology, as well as, the diversity differences observed between COPD and ILD. For example, the increased prevalence of *Haemophilus* compared to *Prevotella* and *Streptococcus* in COPD may contribute to a pro-inflammatory and protease enriched microenvironment that promotes the airflow obstruction by inflating and filing the bronchi with mucus (bronchitis) and/or by destroying extracellular matrix and pulmonary parenchyma (emphysema). Although, we could not establish a link between any taxa and a pro-fibrotic stimulus in ILD, its microbial structure was distinct from that of COPD. The genera *Pseudomonas* and *Staphylococcus* previously described as associated with a worse IPF prognosis^42,52,53^ tended to be higher in our ILD cases compared to COPD and LC*, but this was not significant.

Oddly, although COPD has been shown to increase the risk of LC development 2- to fourfold^21^, the LC* microbiome appears to be more closely related to ILD than COPD. This finding may then question whether the lung microbiome takes part in cancer transformation among COPD patients, particularly when our cases are essentially moderate ones (GOLD 2) and microbial diversity tends to decrease along with disease progression to advanced stages—very severe COPD (GOLD4), reducing the abundances of the potentially carcinogenic genera *Prevotella* and *Veillonella*^9^.

Although less frequently than in COPD, subjects with IPF (ILD) were also reported to be at risk of progressing to cancer^54,55^, suggesting the similarity of LC* and ILD microbiomes as a predisposing factor for cancer occurrence. However, this hypothesis appears to be contradicted by the low prevalence in the ILD group of the cancer associated taxa *Prevotella, Streptococcus* and *Veillonella *^18^*.* On the other hand, the decay of microbial diversity registered from COPD to ILD may be correlated with the severity or life-expectancy of each CLD, in which pulmonary fibrosis tends to have the worse prognosis^17^. In support of this conjecture are former reports of reduced diversity levels in LC and severe COPD and the findings in IPF (ILD) of an association between bacterial burden and patient survival^9,56–58^. If proven true, microbiome studies might be clinically useful to identify patients at risk of cancer complications and to predict disease outcomes.

Even though our study supports previous microbial associations with CLD (e.g., *Haemophilus* and COPD) and provides some evidence for a disease differentiation based in microbiome diversity, it is worth noticing that our comorbidity-controlled groups have a small sample size. Moreover, there is also a large variability in microbiome composition across COPD and LC patients, where some sub-phenotypes (or endotypes) were already connected with specific microbial signatures^9,19,59–61^. Furthermore, in the absence of a healthy group as a control, we could not assess the extent to which the lung microbiome is altered by each CLD. To the best of our knowledge, our work represents a first attempt to consider crossed comorbidities as a factor to characterize the large microbiome heterogeneity in lung cancer cases.

## Conclusions

No clear cut divergence was observed between LC and non-LC cases, aside from the previously recognized *Streptococcus* link to lung cancer. Nonetheless, we uncovered several differences across CLD microbiomes: COPD, ILD and LC* varied not only in microbial composition and evenness, but also in the proportions of *Prevotella* and *Haemophilus.* Altogether, our findings point out to the presence of distinct microbiome hallmarks specific to each CLD subtype that should be further explored in larger cohorts of COPD, LC and ILD cases.

## Supplementary Information

Supplementary Information 1.

Supplementary Information 2.

## Data Availability

The data used in this study is included in the manuscript and in supplementary material files. Sequence data used to generate microbiome analyses is deposited in GenBank under BioProject PRJNA742244.
