# Supplementary material for: Comparative analysis of the bronchoalveolar microbiome in Portuguese patients with different chronic lung disorders
Source: Sci Rep. 2021 Jul 22;11:15042. doi: 10.1038/s41598-021-94468-y (PMC8298389; doi:10.1038/s41598-021-94468-y)
Supplement: Supplementary file 1 — Supplementary Information 1. [file 41598_2021_94468_MOESM1_ESM.pdf]

SUPPLEMENTARY FIGURES

Comparative analysis of the bronchoalveolar microbiome in Portuguese patients with different chronic lung disorders

Susana Seixas*, Allison R. Kolbe, Sílvia Gomes, Maria Sucena, Catarina Sousa, Luís Vaz Rodrigues, Gilberto Teixeira, Paula Pinto, Tiago Tavares de Abreu, Cristina Bárbara, Júlio Semedo, Leonor Mota, Ana Sofia Carvalho, Rune Matthiesen, Patrícia Isabel Marques, Marcos Pérez-Losada

***Corresponding author:** sseixas@ipatimup.pt

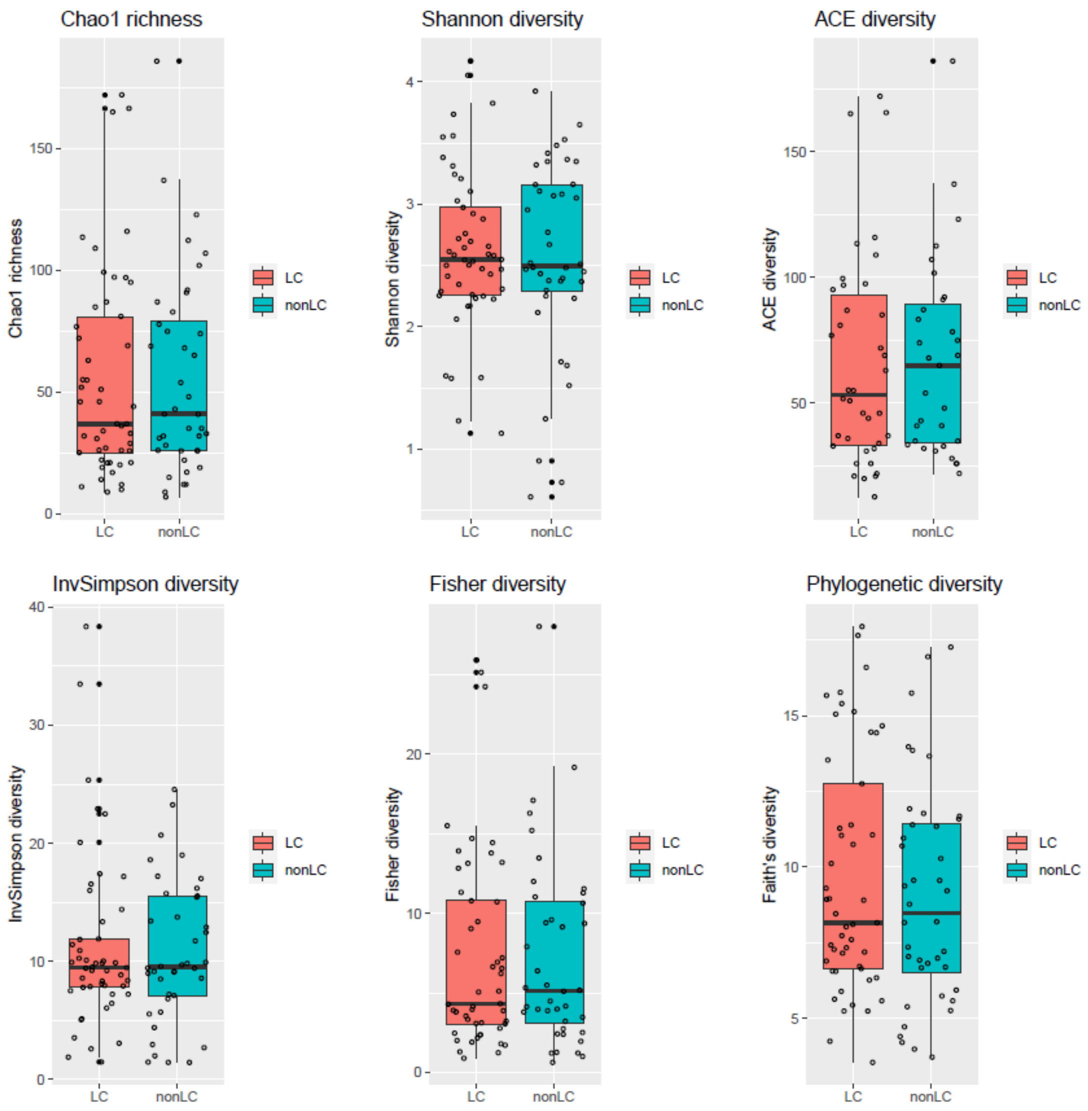

Supp. Figure 1: Alpha diversity of BALF samples for LC (N=49) and Non-LC groups(N=40) Displayed estimates: Chao richness and Shannon, ACE, Inversed Simpson, Fisher and Phylogenetic (Faith's) diversity indices. No significant p-values (<0.05) were obtained.

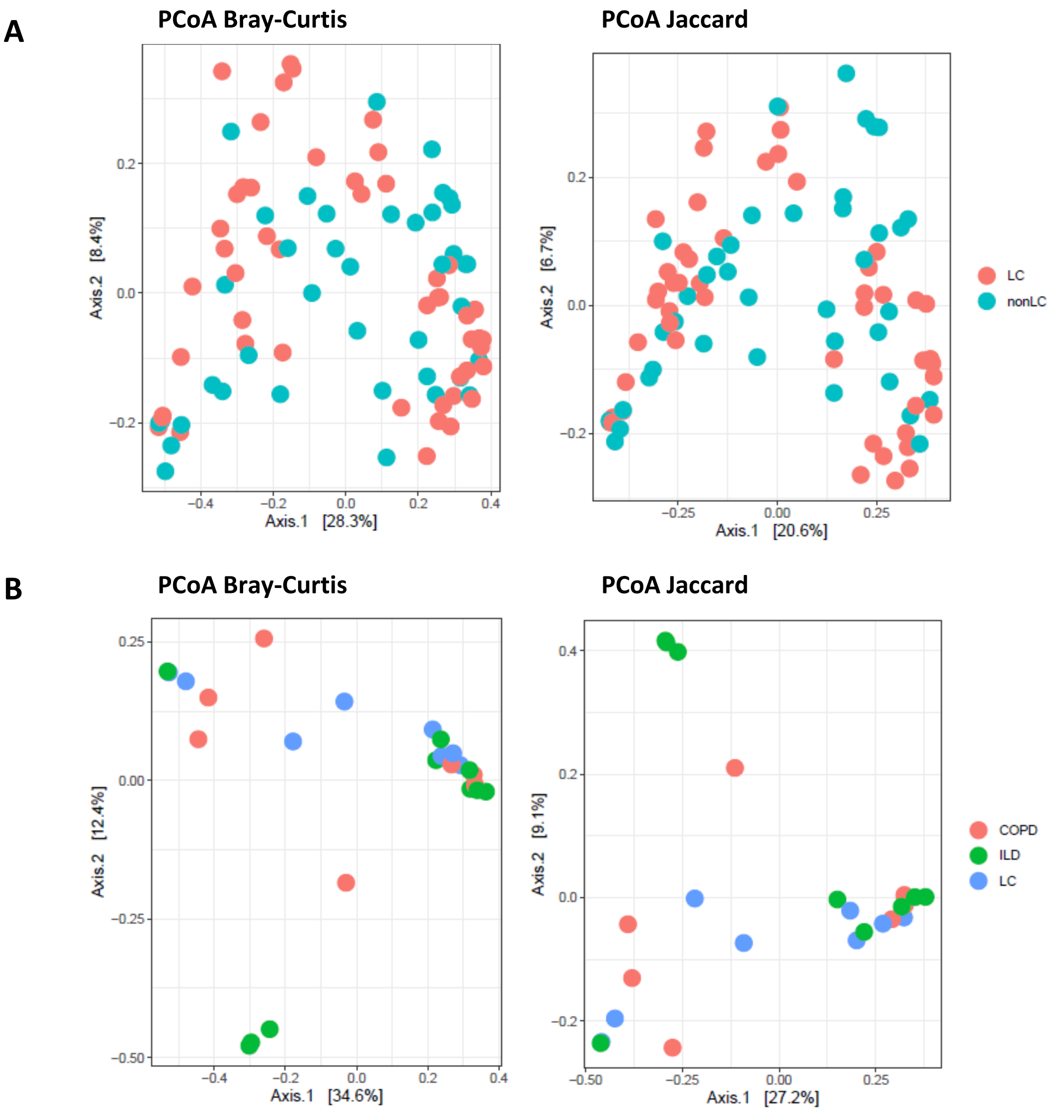

Supp. Figure 2: Beta diversity indices as shown by principle coordinate analysis of Bray-Curtis distances and Jaccard distances. A) Extended dataset comprising LC (N=49) and non-LC groups (N=40); B) Comorbidity controlled dataset including COPD (N=7), ILD (N=10) and LC* (N=8) groups.

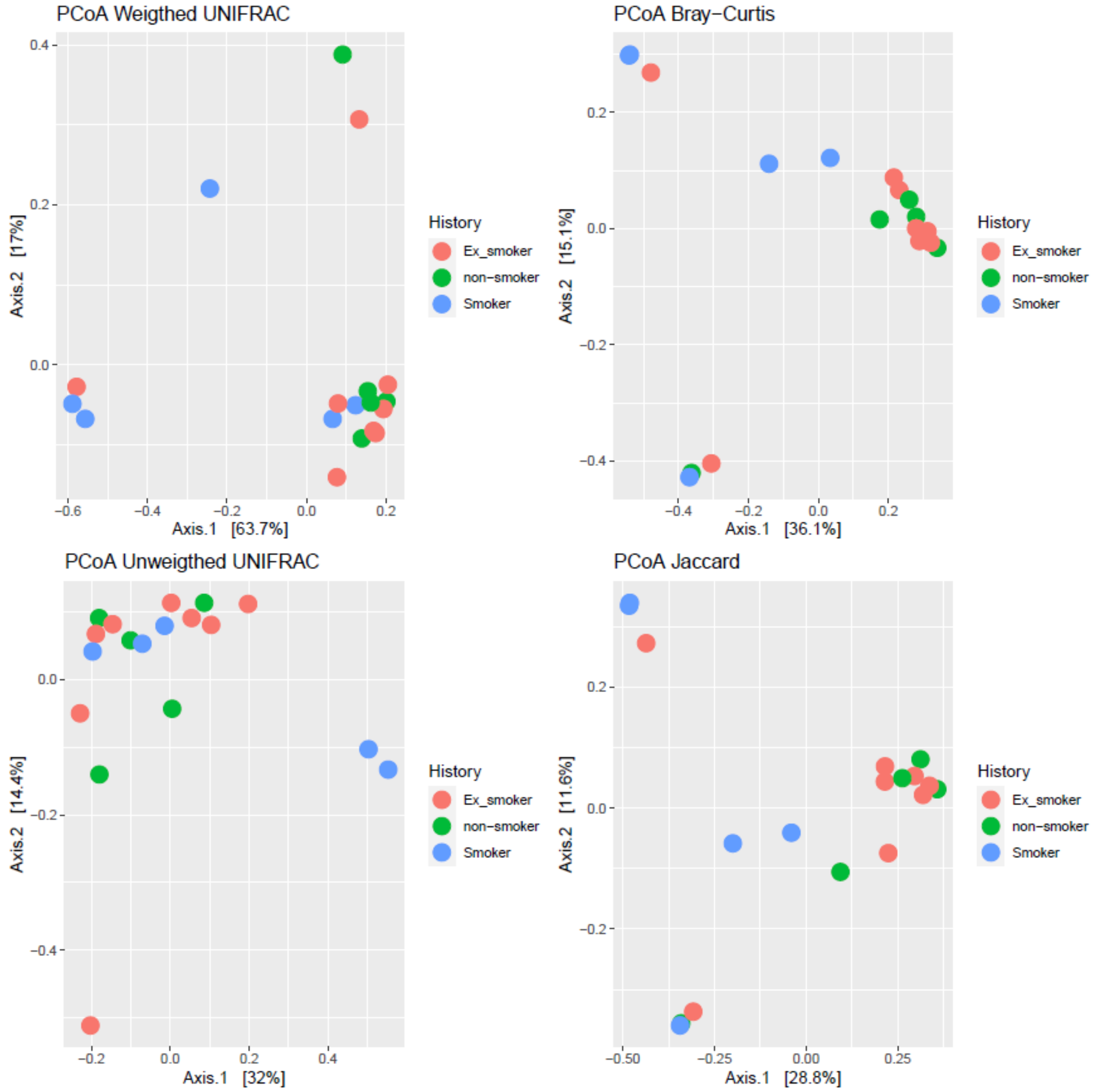

Supp. Figure 3: Beta diversity indices as shown by principle coordinate analysis of weighted UniFrac, unweighted UniFrac, Bray-Curtis (P= 0.027) and Jaccard (P= 0.025) distances obtained for the clinical variable smoking status in LC* (N=8) versus ILD (N=10) comparison.
